# Evaluation of Indirect Fluorescent Antibody Assays Compared to Rapid Influenza Diagnostic Tests for the Detection of Pandemic Influenza A (H1N1) pdm09

**DOI:** 10.1371/journal.pone.0033097

**Published:** 2012-03-30

**Authors:** Sandra Nutter, Michele Cheung, Felice C. Adler-Shohet, Kathryn Krusel, Kate Vogel, Hildy Meyers

**Affiliations:** 1 Epidemiology & Assessment, Orange County Health Care Agency, Santa Ana, California, United States of America; 2 Division of Infectious Diseases, Children's Hospital of Orange County, Orange, California, United States of America; 3 Public Health Laboratory, Orange County Health Care Agency, Santa Ana, California, United States of America; 4 Clinical Laboratory, Saint Joseph Hospital, Orange, California, United States of America; University of Hong Kong, Hong Kong

## Abstract

Performance of indirect fluorescent antibody (IFA) assays and rapid influenza diagnostic tests (RIDT) during the 2009 H1N1 pandemic was evaluated, along with the relative effects of age and illness severity on test accuracy. Clinicians and laboratories submitted specimens on patients with respiratory illness to public health from April to mid October 2009 for polymerase chain reaction (PCR) testing as part of pandemic H1N1 surveillance efforts in Orange County, CA; IFA and RIDT were performed in clinical settings. Sensitivity and specificity for detection of the 2009 pandemic H1N1 strain, now officially named influenza A(H1N1)pdm09, were calculated for 638 specimens. Overall, approximately 30% of IFA tests and RIDTs tested by PCR were falsely negative (sensitivity 71% and 69%, respectively). Sensitivity of RIDT ranged from 45% to 84% depending on severity and age of patients. In hospitalized children, sensitivity of IFA (75%) was similar to RIDT (84%). Specificity of tests performed on hospitalized children was 94% for IFA and 80% for RIDT. Overall sensitivity of RIDT in this study was comparable to previously published studies on pandemic H1N1 influenza and sensitivity of IFA was similar to what has been reported in children for seasonal influenza. Both diagnostic tests produced a high number of false negatives and should not be used to rule out influenza infection.

## Introduction

Accurate and rapid testing of patients for influenza virus is important to optimize antiviral use, minimize antibiotic use, and appropriately isolate hospitalized patients to prevent hospital-acquired infections. During the 2009 H1N1 pandemic, viral culture and real-time reverse transcriptase polymerase chain reaction (rRT-PCR) testing was available through public health laboratories and later through commercial laboratories. However, effective clinical management of patients hospitalized with respiratory illnesses often depends on timeliness of results.

To help with initial diagnosis, many physicians utilized indirect or direct fluorescent antibody (IFA or DFA, respectively) or rapid influenza diagnostic tests (RIDT). Results of RIDT procedures are available within 30 minutes of specimen collection and do not require laboratory expertise but reported sensitivity during the initial stages of the pandemic was low. Several studies looking at RIDT for detection of influenza A(H1N1)pdm09 have shown sensitivity ranging from 18–69% [1]–[11]. IFA and DFA tests are performed in the hospital or reference laboratory and results can be obtained within two to four hours. Additionally, because IFA and DFA tests are usually performed as part of a viral respiratory panel, they are useful for the identification of respiratory viruses other than influenza. Sensitivity of DFA for the detection of A(H1N1)pdm09 has been reported between 47 to 93% [1], [3], [6], [9], [11]–[16]. Information is not currently available on the accuracy of IFA for the detection of A(H1N1)pdm09 in a clinical setting, although a newly developed H1N1-specific IFA claims to have up to 100% sensitivity [17], [18]. More information on the accuracy of IFA tests compared to other diagnostic methods for the detection of A(H1N1)pdm09 is needed to determine their utility in the diagnosis and management of patients with febrile respiratory infections.

## Materials and Methods

### Ethics Statement

During the period of this study, influenza A(H1N1)pdm09 infection was reportable as part of enhanced surveillance in California. The information collected for this study is consistent with activities performed during a public health response and did not require institutional review board approval. Therefore, no consent was obtained, as the specimens included in this study were tested as part of public health surveillance. Technical and physical safeguards to ensure the privacy of protected health information were followed as required by the Health Insurance Portability and Accountability Act of 1996, including maintaining electronic files on secure servers, storing records in locked cabinets and limiting access to authorized personnel.

The Orange County Health Care Agency initiated enhanced surveillance for human cases of pandemic H1N1 on April 24, 2009. Clinicians and community partners were asked to report patients with influenza-like illness meeting certain epidemiologic criteria, which evolved over time based on Centers for Disease Control and Prevention (CDC) and California Department of Public Health guidance, and to submit specimens to the Orange County Public Health Laboratory (OCPHL) for testing. During the initial stages of the 2009 influenza pandemic, surveillance focused on case finding activities. Specimens were accepted at OCPHL if patient had influenza-like illness (ILI), defined as fever ≥100°F, cough and/or sore throat, and met one of the following conditions: (1) had contact with a confirmed case, (2) traveled to areas with pandemic H1N1 activity in the seven days preceding illness onset, (3) had contact with someone with ILI who traveled to areas with pandemic H1N1 activity, (4) had contact with pigs, (5) was part of a defined cluster or outbreak of people with ILI, or (6) was hospitalized with ILI or pneumonia. Submission criterion was revised on June 25, 2010. Patients met the new criteria for testing if they had ILI, pneumonia or severe, unexplained febrile respiratory illness, or sepsis-like syndrome (in infants, adults over 64 years of age, or persons with compromised immune systems) and one of the following: (1) was a health care worker, (2) was pregnant, (3) was part of a defined cluster or outbreak of people with ILI, (4) was hospitalized, (5) or lived in an institutional setting. Testing criteria were revised again on October 2, 2010 to focus on patients who were hospitalized in the intensive care unit or died and had unexplained febrile respiratory illness, ILI, pneumonia or sepsis-like syndrome. Results were included in this analysis if rRT-PCR was performed through OCPHL; both rRT-PCR and either IFA and/or RIDT testing were performed for the same patient; specimens were collected on the same day, and the patient did not have a positive test for seasonal influenza. Results of 638 specimens collected from April 27 through October 14, 2009, from 633 patients met these criteria.

### Specimen types and testing methods

Specimens were received from hospitalized patients (70%), emergency room visits (17%), CDC Sentinel Provider Influenza Surveillance Program sites (10%), and other outpatient visits (3%). Specimen types were known for 600 specimens and included nasopharyngeal or nasal swabs (256; 85%) and washes (322; 54%), tracheal aspirates (18; 3%), bronchoalveolar lavage (3; 0.5%), and lung tissue (1; 0.2%). The majority (84%) of IFA specimens were nasal washes and the majority (68%) of RIDT specimens were nasopharyngeal swabs. Samples were taken at the point-of-care and initial testing was done onsite or referred to a commercial laboratory. Additionally, samples were forwarded to OCPHL for confirmation by rRT-PCR using reagents and protocol provided by CDC (CDC Swine Influenza Virus Real-time rRT-PCR Detection Panel). Each hospital supplied viral transport medium for specimens. Cool packs were used to maintain proper temperature of specimens during transport to OCPHL. Once received by OCPHL, specimens were placed in a refrigerator at 4°C±2°C, then frozen to −70°C prior to extraction. All specimens were typed with InfA and InfB primers and probes. Influenza A positive specimens were sub-typed with seasonal H1 and H3 primers and the CDC Swine Influenza Detection Panel was used to detect swine flu A and swine H1. All IFA testing was conducted at a hospital laboratory serving two hospitals using Bartels® Viral Respiratory Screening and Identification Kit (Trinity Biotech, PLC, Co Wicklow, Ireland). RIDTs were performed at a variety of facilities and included QuickVue Influenza, which does not distinguish between A and B antigens (Quidel Corporation, San Diego, CA), QuickVue Influenza A+B, which distinguishes between A and B antigens (Quidel), and BinaxNOW Influenza A&B test (BinaxNOW; Inverness Medical, Waltham, MA).

Sensitivity and specificity were calculated using rRT-PCR for influenza A(H1N1)pdm09 virus as the reference. Test performance was determined for children (<18 years of age) and adults and for hospitalized patients and outpatients. Data was analyzed using SPSS 16 (SPSS Inc. (IBM), Chicago, IL). Exact Binomial 95% confidence intervals were calculated using JavaStat (http://statpages.org/confint.html), accessed March 2011.

## Results

Results were available for 394 children, 243 adults and 1 person of unknown age. Overall 245 specimens (38%) were positive for influenza A(H1N1)pdm09 (139 children/106 adults). There were 438 respiratory specimens taken from hospitalized patients, of which 131 (30%) were positive for A(H1N1)pdm09 (82 children/49 adults). Of the 200 specimens from non-hospitalized patients, 114 (57%) were positive for A(H1N1)pdm09 (57 children/57 adults). Median age of patients for whom specimens were included was 12 years (range: <1 to 93). The median age for those specimens with confirmed influenza was 15 years (range: <1 to 81).

**Figure 1:**
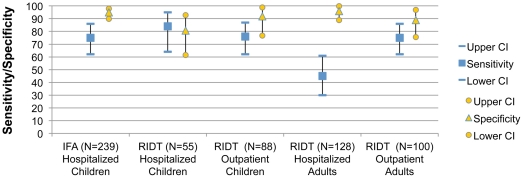
Sensitivity of IFA tests and RIDTs for the detection of pandemic H1N1 influenza. Sensitivity was calculated using real-time reverse transcriptase polymerase chain reaction as the gold standard. IFA results for other groups were not available due to lack of data.

Overall sensitivity of IFA tests and RIDTs was 71% and 69%, respectively. Very few IFA results were received on adults and on outpatient children. Figure 1 and Table 1 presents the sensitivity of RIDT and IFA tests by severity and age. Sensitivity of IFA and RIDT performed on hospitalized children was 75% and 84%, respectively. Sensitivity of RIDT for outpatient children was 76%. In comparison, sensitivity of RIDT performed on adults in outpatient settings was 75% compared to only 45% for hospitalized adults. Overall specificity was 91%. Figure 1 and Table 2 presents the specificity of RIDT and IFA tests by severity and age. Specificity of tests performed on hospitalized children was 94% for IFA and 80% for RIDT. Specificity for RIDT performed in pediatric and adult outpatients was 91% and 90%, respectively. QuickVue Influenza A+B, the most commonly performed RIDT, had a sensitivity of 69% (CI: 60% to 77%) and a specificity of 96% (CI:92% to 98%). Due to small numbers, sensitivity and specificity of QuickVue Influenza (non-A+B) and BinaxNOW A&B RIDTs are not displayed.

**Table 1:** Comparison of Sensitivity for RIDT and IFA Tests by Severity and Age using PCR as the Gold Standard.

		IFA			RIDT		
		Positive	False Negative	Sensitivity (95% CI)	Positive	False Negative	Sensitivity (95% CI)
Inpatients	Children	43	14	75% (62 to 86)	21	4	84% (64 to 95)
	Adults	2	3	----------------	20	24	45% (30 to 61)
Outpatients	Children	2	2	----------------	41	13	76% (62 to 87)
	Adults	—	—	----------------	43	14	75% (62 to 86)

**Table 2:** Comparison of Specificity for RIDT and IFA Tests by Severity and Age using PCR as the Gold Standard.

		IFA			RIDT		
		Negative	False Positive	Specificity (95% CI)	Negative	False Positive	Specificity (95% CI)
Inpatients	Children	171	11	94% (89 to 97)	24	6	80% (61 to 92)
	Adults	9	1	----------------	80	4	95% (88 to 99)
Outpatients	Children	7	1	----------------	31	3	91% (76 to 98)
	Adults	—	—	----------------	38	5	88% (75 to 96)

Mean time from illness onset to specimen collection was similar for hospitalized adults, (2.7 days) and outpatient adults (2.2 days) and was also similar for hospitalized children (2.8 days) and outpatient children (2.1 days), *p*>0.05. Mean age was significantly higher among hospitalized adults (52 years) compared to outpatient adults (31 years), *p*<0.05, and significantly lower among hospitalized children (5 years) compared to outpatient children (9 years), *p*<0.05.

## Discussion

To our knowledge, this is the first study to evaluate an IFA for the diagnosis of influenza A(H1N1)pdm09 in the clinical setting. There is an IFA specifically for the diagnosis of A(H1N1)pdm09 that was approved by the Food and Drug Administration on an emergency use authorization basis, but it has only been evaluated in the lab [17], [18]. In hospitalized children, our IFA performed no better than RIDT. Other investigators looking at DFA and RIDT have had similar findings [9], [16]. Our overall sensitivity of RIDT is comparable to previous published studies on A(H1N1)pdm09 and sensitivity of IFA is similar to what has been reported in children for seasonal influenza (40–90%) when compared to viral culture [19].

In choosing between RIDT and IFA tests, RIDT offers quicker results with similar sensitivity, requires less experienced personnel to perform and utilizes less laboratory personnel time and equipment. However, IFA testing is often performed as part of a respiratory virus panel and positive results for a different respiratory virus than influenza would provide useful information for infection control and other management decisions.

With such a low sensitivity, negative RIDT and IFA test results must be interpreted with caution. Since these tests produce a high number of false negative results, clinicians would not be able to rule out a diagnosis of influenza based on a negative result.

Sensitivity of RIDTs was lower in outpatient children (76%) compared to hospitalized children (84%) and was especially poor in hospitalized adults (45%) compared to outpatient adults (75%). Time from symptom onset to specimen collection was not significantly different between the various groups. We also looked at age as a possible factor affecting sensitivity. Hospitalized children were significantly younger than outpatient children, while hospitalized adults were significantly older than outpatient adults. It is well know that children shed more influenza virus and in greater quantities than adults. However, the effect of age on viral shedding in adults is less established. Clinicians should be aware that sensitivity of RIDTs varies greatly and may be poor in older adults.

The overall specificity of RIDT (91%) is similar to what has been reported in the literature for influenza A(H1N1)pdm09 (86% to 100%), however, results among certain subgroups and test types were much lower than expected [3], [4], [5], [8], [11], [19], [20]. Since RIDT and IFA tests were performed in the clinical setting and PCR testing was performed at a different facility, it is possible that some RIDT and IFA tests were truly positive and the specimens lost integrity during transport to OCPHL. Given that PCR testing performed at OCPHL was used as the gold standard for disease classification, this would result in more false positives then is accurate due to misclassification of those who had A(H1N1)pdm09. While our results may be due to small sample size or loss of specimen integrity during transport, healthcare providers should be aware that these tests may produce false positives under certain conditions.

Our study is limited by the lack of detailed information recorded on specimen type (i.e. nasal swab versus nasopharyngeal swab) restricting our ability to account for different collection methods in our analysis. Additionally, in a small number of patients, the specimen tested by IFA or RIDT may not have been the exact same specimen tested by rRT-PCR although all specimens were collected on the same day. One hospital laboratory performed all IFA testing, while RIDT testing was performed in a variety of facilities and using different methods. Finally, since IFA and RIDT were performed at a different facility than rRT-PCR, storage or transportation issues (including transport temperature) may have affected the integrity of the sample and are limitations of the study.

In our study, clinically based diagnostic tests for influenza A(H1N1)pdm09 had variable sensitivities and specificities and may lead to false negative and false positive results. Treatment and infection control decisions should not be changed or delayed based on negative IFA or RIDT results. Research is needed to develop and validate more sensitive rapid testing for influenza.
